# Physical Exercise with or without Whole-Body Vibration in Breast Cancer Patients Suffering from Aromatase Inhibitor—Induced Musculoskeletal Symptoms: A Pilot Randomized Clinical Study

**DOI:** 10.3390/jpm11121369

**Published:** 2021-12-14

**Authors:** Alessandro de Sire, Lorenzo Lippi, Antonio Ammendolia, Carlo Cisari, Konstantinos Venetis, Elham Sajjadi, Nicola Fusco, Marco Invernizzi

**Affiliations:** 1 Physical and Rehabilitative Medicine Unit, Department of Medical and Surgical Sciences, University of Catanzaro “Magna Graecia”, 88100 Catanzaro, Italy; ammendolia@unicz.it; 2 Physical and Rehabilitative Medicine, Department of Health Sciences, University of Eastern Piedmont, 28100 Novara, Italy; cisari50@gmail.com (C.C.); marco.invernizzi@med.uniupo.it (M.I.); 3 Department of Oncology and Hemato-Oncology, University of Milan, 20122 Milan, Italy; konstantinos.venetis@unimi.it (K.V.); elham.sajjadi@unimi.it (E.S.); nicola.fusco@unimi.it (N.F.); 4 Division of Pathology, IEO, European Institute of Oncology IRCCS, 20144 Milan, Italy; 5 Translational Medicine, Dipartimento Attività Integrate Ricerca e Innovazione (DAIRI), Azienda Ospedaliera SS, Antonio e Biagio e Cesare Arrigo, 15121 Alessandria, Italy

**Keywords:** breast cancer, precision medicine, aromatase inhibitors, pain, physical exercise, whole-body vibration, quality of life

## Abstract

In this study, we aimed to assess the safety and efficacy of physical exercise, with or without whole-body vibration (WBV), in patients with aromatase inhibitor-induced musculoskeletal symptoms (AIMSS). Eligible patients were adults (≥18 years) with a history of breast cancer and current AIMSS. Enrolled patients (*n* = 22) were randomly assigned 1:1 to receive physical exercise combined with WBV or sham WBV for 4 weeks. The primary endpoint was pain intensity measured by numerical pain rating scale (NPRS). The secondary endpoints were muscle strength, physical function, physical performance, and quality of life. The WBV group (mean age: 51.73 ± 10.73 years; body mass index (BMI): 25.56 ± 5.17 kg/m^2^) showed a statistically significant pain reduction (NPRS: 6.82 ± 1.17 vs. 5.73 ± 1.01; *p* = 0.031), whereas patients in the sham WBV group (mean age: 58.55 ± 9.71 years; BMI: 27.31 ± 3.84 kg/m^2^), did not reach statistical significance (NPRS: 6.91 ± 2.02 vs. 5.91 ± 2.51; *p* = 0.07). Concurrently, muscle strength, physical performance, and quality of life significantly improved in both groups, without significant differences between groups. No dropouts and no side effects were recorded. Both patients and the physical therapist reported a high level of satisfaction with the intervention. Our findings suggest that physical exercise and WBV combination might be a safe therapeutic option for improving the rehabilitative management of patients with AIMSS.

## 1. Introduction

The improvement of health-related quality of life (HRQoL) in women with a history of breast cancer has become a high priority due to the increasing number of survivors [1,2]. Recently, it has been highlighted that a tailored assessment of HRQoL should involve physical, psychological, and emotional well-being, which might be significantly affected by anxiety, fatigue, lymphedema, pain, and menopausal symptoms [3].

Aromatase inhibitors (AIs) interfere with the body’s ability to generate estrogen from androgens by inhibiting the action of the aromatase enzyme [4]. Due to their effectiveness in hormone receptor (HR)-positive breast cancers, AIs represent a backbone therapy, particularly in postmenopausal women [5]. However, AIs administration has been related to common side effects that represent a critical issue in the current literature [6,7]. Moreover, to date, the mechanisms forming the basis for the onset of side effects are far from being fully understood but might be related to the severe estrogen depletion [7]. Among these, AIs-induced musculoskeletal symptoms (AIMSS) are an often underdiagnosed and underestimated complication of this type of treatment [8]. This condition is defined by the presence of painful musculoskeletal disorders during endocrine therapy with AIs [9]. It has recently been estimated that up to 40% of HR+ breast cancer survivors treated with AIs experience AIMSS [10,11,12]. Regrettably, due to such complications, the discontinuation rate of AIs could reach 20% within the first year of therapy [12,13,14,15]. Furthermore, AIMSS significantly affects the HRQoL social life, with dramatic effects on women’s socioeconomic life and subsequent psychological frailty [16]. Therefore, the management of AIMSS is a foremost clinical need [8].

**Figure 1:**
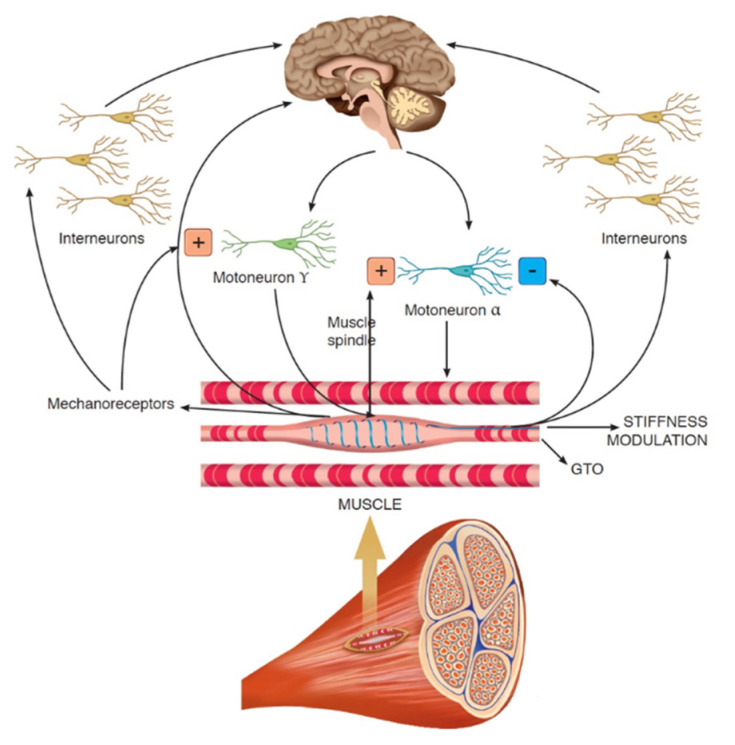
The role of whole-body vibration on neuromuscular fuses.

Physical exercise (e.g., aerobic and resistance training), nutritional supplements (e.g., glucosamine plus chondroitin and vitamin D), and acupuncture have been applied in many centers for pain management in patients with breast cancer and breast cancer survivors, including those with AIMSS [8,17,18,19,20,21]. Furthermore, there has been growing interest regarding the use of physical therapy with whole-body vibration (WBV) training for pain management in patients dealing with musculoskeletal disorders [22,23,24]. In particular, WBV is effective neuromuscular training that uses various frequencies of mechanical vibration to induce repetitive muscle contractions to enhance muscle function and proprioception [25]. This type of physical therapy has been widely used to improve muscle strength and performance [26,27]. The beneficial effect of WBV is typically ascribed to the tonic vibration stretch reflex, a specific involuntary reflex mechanism induced by the fast changes in the length of the muscle-tendon complex (see Figure 1) [28,29]. To date, WBV has been studied in the management of both acute and chronic musculoskeletal pain in patients with low back pain and women with fibromyalgia, reporting intriguing implications in HRQoL [30,31,32].

Moreover, WBV has been recently proposed to improve bone turnover and functional outcomes in cancer patients, with controversial findings [22,33].

The role of physical exercise has been scarcely explored in AIMSS, with contradictory results [31,34]. Furthermore, studies of tailored protocols including WBV training are lacking for these patients. In this pilot randomized study, we sought to investigate the feasibility and effectiveness of physical exercise combined with WBV for the clinical management of AIMSS.

## 2. Materials and Methods

### 2.1. Study Design and Patients

This pilot randomized clinical study involved a consecutive series of breast cancer patients suffering from AIMSS. These patients were referred to the Cancer Rehabilitation Center in the Hospitals of the University of Eastern Piedmont, Novara-Alessandria, Italy, over 6 months, from June 2020 to November 2020. Inclusion criteria were: (a) diagnosis of HR+ breast cancer; (b) AIMSS onset during endocrine therapy with AIs; (c) women aged more than 18 years; (d) 1-week wash-out from oral analgesic drugs and NSAIDs; (e) written, dated and signed informed consent. The exclusion criteria were: (a) metastatic breast cancer; (b) concurrent chemotherapy; (c) cognitive impairment; (d) fractures in the past 6 months; (e) presence of pacemaker and/or electrical implantable devices; (f) other absolute contraindications to physical activity. All of the participants were enrolled after the end of surgical therapy, radiotherapy, and/or chemotherapy. Moreover, the AIMSS diagnosis had been performed by a clinician with years of expertise in cancer rehabilitation.

This study was approved by the local Institutional Review Board (n.677/2020) and was compliant with the ethical guidelines of the responsible governmental agency. At the enrollment, all the participants were asked to carefully read and sign an informed written consent. The investigators provided to protect the privacy and the study procedures following the Declaration of Helsinki [35].

### 2.2. Randomization and Procedures

After enrollment, all patients were divided through a randomization scheme with a 1:1 allocation to either a real WBV arm (Group A) or sham WBV arm (Group B). All of the patients underwent an exercise protocol, consisting of a 4-week semi-standardized rehabilitative treatment protocol. The duration of each session ranged from 50 to 60 min, three times per week, with at least 1 day of rest between each session.

**Figure 2:**
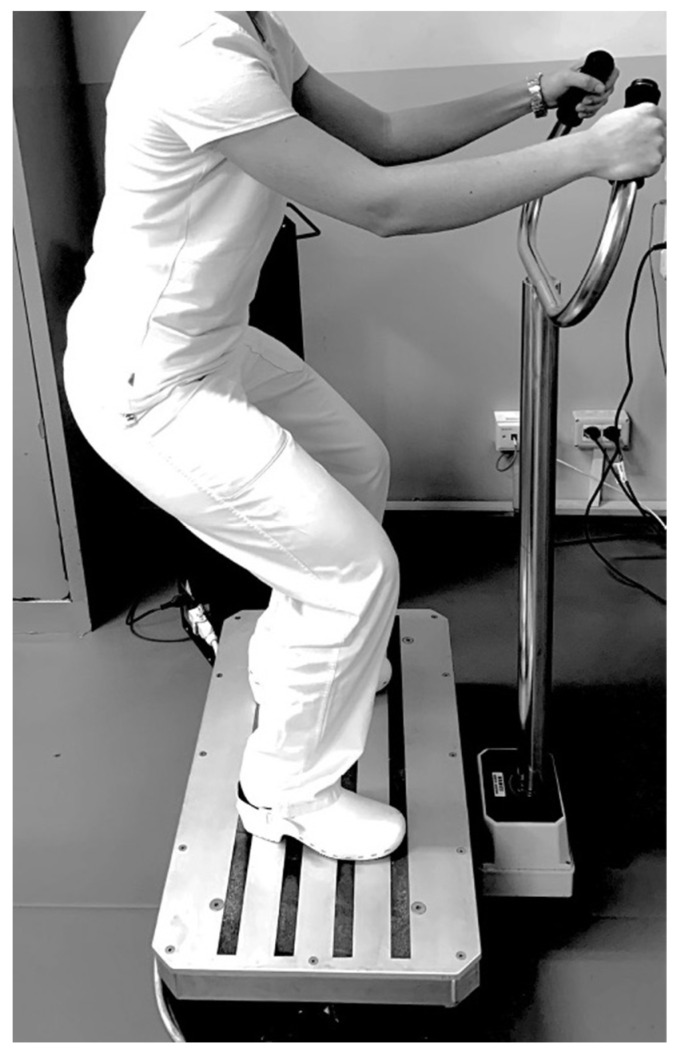
Example of physical exercise plus whole-body vibration.

The semi-standardized rehabilitative treatment protocol consisted of 5 phases:

- Phase I (common for both groups, lasting 10 min): a warm-up characterized by active and passive joint mobility exercises, performed to adapt the joints to the load and reduce the rate of musculoskeletal or other injuries.
- Phase II (common for both groups, lasting 10 min): aerobic training on treadmill (rapid walking at 3–5 km/h) with exercise intensity set according to 60–70% of maximal heart rate.
- Phase III (with differences between groups, lasting 10 min): a resistance exercise training protocol comprising both isotonic and isometric exercise, with intensity assessed by the Modified Borg Scale (MBS) [36]. The target of exercise intensity was set to moderate with an MBS score between 4 and 6. Moreover, all patients were encouraged to reach the target intensity during the training session. In this phase, the two groups performed the physical exercise with or without the WBV approach with the following differences: Group A (physical exercise plus WBV) performed 5 sets of 10 repetitions of squats without WBV alternating with 5 sets of 30 s on the side-alternating WBV platform (model NEMES-LB Bosco System^®^ Rieti, Italy), with a frequency of 30 Hz, peak-to-peak amplitude of 1.15 mm (acceleration magnitude of 20.44 ms^–2^) in squatting position (110° knee flexion), as shown in Figure 2.

Group B (physical exercise plus sham WBV) performed 5 sets of 10 repetitions of squats without WBV alternating with 5 sets of 30 s on WBV platform (model NEMES-LB Bosco System^®^, Rieti, Italy), with sham treatment and no frequency (0 Hz) in an isometric squat (110° of knee flexion).

- Phase IV (common for both groups, lasting 10 min): equal to Phase II consisting of aerobic training on treadmill (rapid walking at 3–5 km/h) with exercise intensity set according to 60–70% of maximal heart rate.
- Phase V (common for both groups, lasting 10 min): cooling down with active and passive joint mobility exercises.

Participants in both groups wore sports shoes during the training sessions. Each treatment session was supervised by the same physiotherapist with expertise in cancer rehabilitation.

### 2.3. Outcome Measures

At the baseline (T0), anamnestic, demographic, and clinical characteristics were assessed. The type of breast and axillary surgery, previous treatments (e.g., radiotherapy, hormone therapy, and trastuzumab), and presence of upper limb lymphedema were recorded.

The primary outcome measure of this study was the modification of pain intensity, measured by the Numerical Pain Rating Scale (NPRS) in the two groups. Secondary outcomes were: appendicular muscle strength assessed by hand grip strength test (HGS) [37] using the Jamar hydraulic hand dynamometer (Sammons Preston, Rolyon, Bolingbrook, IL, USA); the Western Ontario and McMaster Universities Osteoarthritis Index (WOMAC) to assess pain, joint stiffness and function [38]; 10 m walking test (10MWT) [39] and 6MWT to evaluate physical performance [40]; European Organization for Research and Treatment of Cancer Quality of Life Questionnaire (EORTC QLQ-C30) [41] and its subscales (functional scale, symptom scales, and global health scale).

All of the outcome measures were assessed at both T0 and after 1 day of rest from the last treatment session at the end of the 4-week rehabilitative treatment (T1) by a provider relationship management (PRM) physician, blinded to the treatment group.

Furthermore, the safety of WBV was evaluated through a precise assessment of side effects after each treatment session. Lastly, both patients and the physical therapist involved in this study expressed their satisfaction regarding the intervention at the end of treatment (T1), through the global perceived effect (GPE) scale, ranging from 1 (best satisfaction) to 7 (unsatisfaction) [42].

### 2.4. Statistical Analysis

Statistical analysis was performed using GraphPad Prism 7.0 (GraphPad Software, Inc., San Diego, CA, USA). Categorical variables were represented as numbers and ratios, whereas continuous variables were described as means ± standard deviations. Due to the small sample size, we assumed a non-Gaussian distribution of variables. Intra-group differences for each variable were evaluated by two-tailed Wilcoxon’s signed-rank test, whereas between-group analysis was performed by two-tailed Mann–Whitney U test. A *p*-value lower than 0.05 was considered as statistically significant. Descriptive statistics were used to summarize the adverse effect of the treatment.

## 3. Results

Out of 36 breast cancer patients assessed for eligibility, 14 were excluded (11 did not meet inclusion criteria, 3 declined to participate). Thus, a total of 22 study participants were enrolled and randomly allocated into 2 groups: Group A (*n* = 11; mean age: 51.73 ± 10.73 years; body mass index (BMI): 25.56 ± 5.17 kg/m^2^) and Group B (*n* = 11; mean age: 58.55 ± 9.71 years; BMI: 27.31 ± 3.84 kg/m^2^).

**Figure 3:**
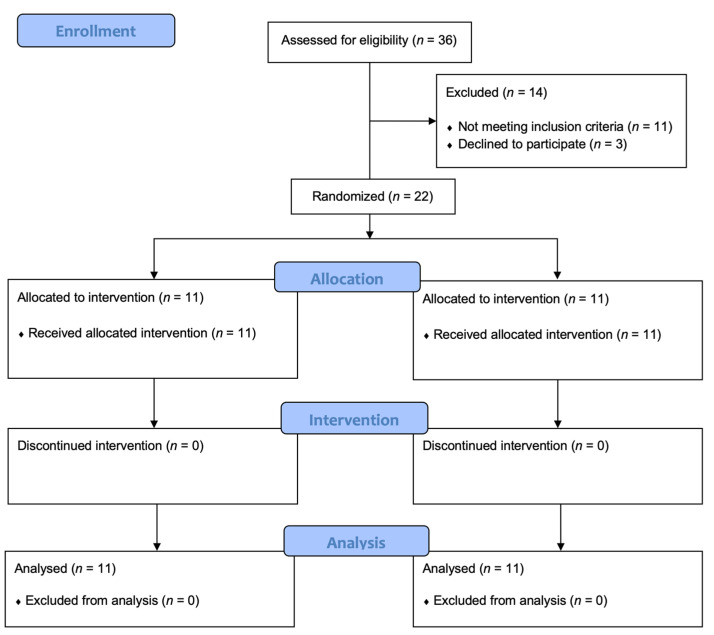
Study flow-chart.

The study flow chart is depicted in Figure 3.

There were no statistically significant differences between the groups in terms of age, BMI, breast surgery, axillary surgery, radiotherapy, or upper limb lymphedema. Baseline characteristics of patients are reported in Table 1.

Concerning the primary outcome, Group A patients showed a statistically significant pain reduction (NPRS: 6.82 ± 1.17 vs. 5.73 ± 1.01; *p* = 0.031), whereas patients in Group B did not reach statistical significance in terms of pain relief (NPRS: 6.91 ± 2.02 vs. 5.91 ± 2.51; *p* = 0.07). However, there were no significant differences between groups in primary outcome at T1 evaluation. Both groups showed a statistically significant improvement in functioning, assessed by WOMAC, and a significant difference between groups at T1 (Group A: 77.56 ± 9.853; Group B: 65.63 ± 14.27; *p* = 0.044), suggesting a greater improvement in functioning in Group A patients.

Concurrently, muscle strength (assessed by HGS), physical performance (6 MWT and 10 MWT), and HRQoL (EORTC QLQ-C30) significantly improved in both groups, without significant differences between groups (see Table 2 for further details on primary and secondary outcomes). During the whole study period, no dropouts in either group were recorded. Moreover, all enrolled patients showed no side effects. Only one patient (4.6%) in Group A reported nausea after the first session of physical exercise plus WBV. However, this symptom was self-limited and did not prevent the patient from performing the rest of the rehabilitative sessions. Lastly, both patients (mean GPE score at T1 were 2.2 for Group A and 1.9 for Group B) and the physical therapist (GPE score = 2.0 at T1) reported a high level of satisfaction with the rehabilitative intervention performed.

## 4. Discussion

This pilot randomized clinical study represents the significant effects of a multidimensional rehabilitative intervention comprising both physical exercise and WBV in reducing pain and improving muscle strength, functioning, physical performance and HRQoL in breast cancer patients affected by AIMSS. Our findings are in line with previous findings regarding the positive role of exercise in AIMSS management [12,43]. We aimed at expanding this knowledge by combining the positive role of exercise with the implication of WBV. Indeed, we noticed a significant reduction in NPRS only in the study group (not in the control group). On the other hand, similar reductions in terms of pain relief were reported, and the comparison of T0 and T1 between the two groups showed no significant differences.

However, significant improvements in physical performance, muscle strength, and HRQoL in both the study and the control groups were seen. These significant improvements in secondary outcomes in both groups supported previous evidence suggesting positive effects of physical activity in breast cancer survivors with AIMSS.

Intriguingly, HST significantly improved in both groups even without a specific isometric strength training for hand muscles. However, these results are in line with previous findings supporting the positive effects of whole-body muscle training in HST [44,45]. The mechanisms underlying these increases are far from being fully understood; however, it has been proposed that training-induced neuromuscular adaptations and systemic response to muscle training might have a role in the improvement of handgrip muscle force [46].

Moreover, the significant difference between groups in WOMAC scores (*p* = 0.044) at the end of treatment supports our hypothesis that the combination of physical exercise with WBV could provide greater functioning and strength improvements in these women compared to traditional rehabilitative interventions.

Furthermore, we provide further credence to the effects of WBV in reducing pain in other musculoskeletal conditions such as low back pain, knee osteoarthritis, peripheral neuropathies, and fibromyalgia [47,48,49,50,51]. However, despite these promising results in different pathological conditions, the precise mechanism of action underpinning pain relief induced by WBV is still far from being understood in detail. An intriguing physiological hypothesis concerns the gate control theory proposed by Melzack and Wall in 1962 [49]. More in detail, vibration therapy could promote the activation of low threshold mechanoreceptors that are vibration receptors, such as Merkel’s disk, Meissner’s corpuscle, and Pacinian corpuscle [50,51,52]. In this context, the prolonged and repeated afferent stimuli could promote substantial gelatinase activation through the nervous fiber type Aβ, triggering local inhibitory circuits in the dorsal horn of the spinal cord. Therefore, the nociceptive afferent stimuli transduced by C fibers might, as a result, be inhibited by presynaptic stimuli induced by the vibrations [23,52,53,54]. Furthermore, another mechanism potentially responsible for pain relief induced by WBV could be related to the central nervous system mechanism involved in chronic pain sensation. Jamal et al. [55] suggested that vibration might influence the cerebral cortex area receiving pain sensation by direct facilitation, based on the anatomical proximity of the pain sensation area to vibrotactile perception [56].

We showed that physical exercise plus WBV seems to be safe, well-tolerated (no dropouts in either group during the entire treatment), and high satisfaction was reported by both the patients and the health personnel involved in the study. Therefore, our findings point to the need to clearly define a precise rehabilitative intervention, including physical exercise and WBV, tailored for breast cancer survivors with AIMSS (Figure 3). To the best of our knowledge, this is the first study evaluating the efficacy of exercise combined with WBV for pain relief in breast cancer patients affected by AIMSS. Our results suggested promising features of the use of WBV as an additional tool in the rehabilitation of patients with AIMSS.

We acknowledge that further studies, comprising a larger cohort of patients and longer follow-up data, are required to support our findings.

### Study Limitations

We are aware that this study is not free from limitations. First, the small sample size, due to the strict eligibility criteria, did not allow us to draw strong conclusions. However, it should be noted that in accordance with the pilot design, we aimed to assess the feasibility of the WBV intervention in a small-scale study to provide preliminary data for a new intervention in this field. Second, despite no differences between groups being underlined at baseline, the small sample size might have affected these results. Third, longer follow-up might be interesting for better understanding of the role of WBV combined with physical exercise.

## 5. Conclusions

In conclusion, our findings regarding pain do not convincingly show differences between physical exercise with or without WBV. However, the combination of WBV with physical exercise might be considered a safe and well-tolerated intervention to reduce pain and disability while enhancing AIMSS patients’ HRQoL. Future studies are warranted to understand the role of WBV in BC survivors, assessing wider cohorts with longer follow-ups to improve knowledge about the most effective therapeutic and rehabilitative strategies to manage AIMSS in breast cancer patients.

## Figures and Tables

**Table 1:** Anamnestic, demographical, and clinical characteristics of study population.

Sample Characteristics	Group A (*n* = 11)	Group B (*n* = 11)	*p* Value
Age (years)	51.73 ± 10.73	58.55 ± 9.71	0.146
Sex (female)	11 (100.0)	11 (100.0)	0.999
BMI (kg/m^2^)	25.56 ± 5.17	27.31 ± 3.84	0.186
Smokers (habitual smokers)	2 (18.2)	1 (9.1)	0.999
Breast surgery			
Conservative	6 (55.5)	5 (44.5)	0.999
Mastectomy	5 (44.5)	6 (55.5)	0.999
Axillary surgery			
Sentinel lymph node	5 (36.4)	5 (44.5)	0.999
En bloc dissection	7 (63.6)	6 (55.5)	0.999
Radiotherapy	8 (72.7)	6 (55.5)	0.659
Hormone therapy	11 (100.0)	11 (100.0)	0.999
Chemotherapy	4 (36.4)	3 (27.3)	0.999
Trastuzumab	3 (27.3)	4 (36.4)	0.999
Presence of Upper Limb Lymphedema	0 (0.0)	1 (9.1)	0.999

Continuous variables are expressed as means ± standard deviations, categorical variables are expressed as counts (percentages). Abbreviations: BMI: Body Mass Index.

**Table 2:** Intra-group and between-group differences in outcome measures in the two groups.

Outcome	Group A (*n* = 11)			Group B (*n* = 11)			Between-Group Analysis	
	T0	T1	*p*-Value	T0	T1	*p*-Value	T0 *p*-Value	T1 *p*-Value
NPRS	6.82 ± 1.17	5.73 ± 1.01	0.031	6.91 ± 2.02	5.91 ± 2.51	0.070	0.430	0.434
WOMAC	52.69 ± 13.21	77.56 ± 9.85	0.001	59.12 ± 19.71	65.63 ± 14.27	0.023	0.508	0.044
HGS	15.81 ± 0.59	17.42 ± 1.06	0.001	13.24 ± 2.13	15.36 ± 3.37	0.016	0.077	0.185
6MWT	465.51 ± 57.94	518.2 ± 44.79	0.017	423.7 ± 45.39	470.9 ± 57.52	0.001	0.063	0.186
10MWT	1.46 ± 0.26	1.61 ± 0.17	0.001	1.42 ± 0.16	1.60 ± 0.16	0.001	0.885	0.884
EORTC QLQ-C30								
Functional score	69.74 ± 12.42	80.61±10.14	0.027	70.71 ± 21.77	78.99 ± 16.28	0.004	0.736	0.783
Symptom score	31.72 ± 12.26	19.35 ± 7.46	0.008	28.44 ± 12.48	20.05 ± 11.46	0.001	0.548	0.505
Global health score	25.76 ± 13.67	60.61 ± 17.91	0.001	34.09 ± 9.47	68.18 ± 22.61	0.005	0.190	0.422

Continuous variables are expressed as means ± standard deviations. *p* values are considered significant when *p* is less than 0.05. Abbreviations: T0: Baseline; T1: After rehabilitation; 6MWT: 6-min walking test; 10MWT: 10-min walking test; EORTC QLQ-C30: European Organization for Research and Treatment of Cancer Quality of Life Questionnaire; HGS: hand grip strength test; NPRS: numerical pain rating scale; WOMAC: Western Ontario and McMaster Universities Osteoarthritis Index.

## Data Availability

The datasets generated and/or analyzed during the current study are available from the corresponding author on reasonable request.
